# Paralysis of the Facial Nerve

**Published:** 1881-06

**Authors:** J. J. Caldwell

**Affiliations:** Baltimore, Md.


### ARTICLE III.

### *Paralysis of the Facial Nerve.*

BY J. J. CALDWELL, M. D., BALTIMORE, MD.

This nerve, the seventh pair, leaves the sides of the medulla oblongata, just behind the pons varolie. Its fibres are traced to a collection of gray matter in the upper part of the medulla, continuous with that from which the roots of the sixth pair originate. After leaving the side of the medulla it passes onward through the petrous portion of the temporal bone, emerges at the stylo-mastoid foramen, bends round beneath the external ear, and passes forward through the substance of the parotid gland, forming a plexus called the "pes anserinus," by the abundant inosculation of its different branches. It then sends its filaments forward, in a diverging course, and is finally distributed to the muscles of the external ear, to the frontalis and superciliaris muscles, to the orbicularis oculi, the compressors and dilators of the nares, the orbicularis oris, and to the elevators and depressors of the lips; that is, to the superficial muscles of the face, which are concerned in the production of expression. The facial is the motor nerve of the face. It has nothing to do with transmitting sensitive impressions, since it has been frequently shown that after section of the fifth pair, the facial remaining entire, the sensibility of the face is completely lost; so that the integument may be cut, pricked, pierced, or lacerated, without any sign of pain being exhibited by the animal. The facial, therefore, does not transmit sensation from these parts; and its division which was formerly resorted to in cases of tic douleureux, is accordingly altogether incapable of relieving neuralgic pains.

This nerve, however, is directly connected with muscular action, since mechanical or galvanic irritation of its fibres produces convulsive twitching in the ears, nostrils, lips, and cheeks. According to Dalton the division of the facial, in one of the lower animals, (such as the cat,) just after its emergence from the stylo-mastoid foramen will result in complete muscular paralysis of those parts to which the nerve is distributed, while the power of sensation remains unimpaired. There is incapability of moving the ear, which maintains a rigid position. There is also incapacity of closing the eyelids on account of paralysis of the orbicularis oculi, and the eye consequently remains open, even when the other eye is closed, during sleep or in the act of winking. If the conjunctiva be touched, there is an immediate appreciation of it; the eyeball is drawn partially backward into the socket by the action of the recti muscles. Precisely opposite effects are produced upon the eyelids by paralysis of the oculo-motorius nerve, and by that of the facial. In the former instance, owing to the paralysis of levator palpebræ superioris, the eye is always partially closed; in the latter, owing to paralysis of the orbicularis, it is always partially open. There is also a suspension of the movements of the nerves on the side of the injury, and a depression of the angles of the mouth on same side. The phenomenæ of the division of the facial nerve differentiates in various animals; for example, in the rabbit, the ear upon the affected side droops, and its movements are beyond the control of the animal.

In the horse the division of the facial nerve eventuates in death by suffocation, on account of the animal breathing exclusively through the nostrils.

In the human subject the facial nerve is, from various causes, occasionally paralyzed on one side. This effect is known as "facial paralysis," in which there is an entire absence of expression on the affected side of the face, the lower portion of which is drawn to the opposite side by the force of the unaffected antagonistic muscles. In facial paralysis the principal inconvenience depends upon labial muscular action, and the muscles about the cheek. Owing to the paralysis of the buccinator muscle, which receives its motor filaments from the facial nerve, fluids, in drinking, escape by the corner of the mouth, and, measurably, food, in the act of mastication.

The fact of animals showing evidences of painful sensations, when the branches of the facial are irritated, indicates that these filaments have a certain degree of sensibility, though the facial may be regarded only as a motor nerve.

"Longet," says Dalton, "has shown by an extremely ingenious mode of experiment that the sensibility of the branches of the facial does not depend on any sensitive fibres of their own, but upon those which they derive *from inosculation with the fifth pair.*" The general conclusion therefore, is that a certain degree of sensibility is communicated to the branches of the facial by filaments derived from the fifth pair.

The above facts, says Dalton, "account for the peculiar circumstance that, in cases of tic douleureux, the spasmodic pain sometimes follows exactly the course of the facial nerve, viz: from behind the ear forward upon the side of the face; and yet the section of this nerve does not put an end to the neuralgia, but only causes paralysis of the facial muscles." The aforegoing, or facial paralysis, is often known as Bell's paralysis, on account of its real nature, having been first clearly pointed out by Sir Charles Bell. The paralysis of the occipito-frontalis and of the corrugator supercilii prevents the raising of the eyebrows, or frowning and obliterates all wrinkles from the brow. As Romberg frocetiously remarks, "there is no better cosmetic for elderly ladies than facial paralysis."

Diagnosis.—Facial paralysis, writes Hammond, "is distinguished from glosso-labio-laryngeul paralysis, by the facts that in the latter the symptoms affect only the lower part of the face, and that they are accompanied by paralysis of the tongue and of the muscles of deglutition. From the facial paralysis of hemiplegia it is diagnosticated by the marked circumstance that, in the latter disorder, the patient can close the eye, while in the former it remains wide open. There are no other affections with which facial paralysis can be confounded if the slightest attention be given to its symptoms."

According to Althaus, most cases of facial palsy are due to the influence of damp and cold. A rheumatic effusion takes place into the cellulor tissue of the face, by which the peripheral branches of the portio dura are compressed, and their function more or less inhibited.

Cases which occur in children or young persons, and where the quantity of lymph effused is not very great, may get well spontaneously; but in adults, or where a large effusion has taken place, the palsy only yields to appropriate treatment. Where all the muscles of the face are equally effected, the nerve is, as a rule, compressed by an effusion in the fallopian canal. In slight cases of this kind the fasado-muscular excitability is sometimes increased and sometimes normal, but only in exceptional cases diminished or lost.

The treatment generally in these cases consists of blistering, and the internal use of iodide of potassium or strychnia. Electricity is, however, much more rapidly successful, especially if the affection be of recent origin. Many cases may be cured by faradisation; but where this fails, and likewise in those cases where forado-muscular contractility is altogether lost, galvanization is preferable. Faradism should be applied to all the paralyzed muscles individually; while if the constant current be used, the anode is placed to the auriculo-maxillary fossa, and the cathode gently passed over the peripheral branches of the portio dura. Voltaic alternations are useful; where the external application does not produce much benefit, the negative pole may be applied to the mucous membrane of the cheek, and the positive externally, which sometimes does good, after all other modes of applying electricity have failed.

Cases of rheumatic paralysis of the portio dura generally yield to treatment, even if they have existed for a very long time.

Professor Ore has related a case of eight and a half years duration, which was cured by farodism, and Dr. Russell Reynolds succeeded by the same means in notably improving one of fourteen years standing. Facial palsy is also observed as a symptom of cerebral hemiplegia, but it then generally appears only in a few muscles of the face; viz: the *levator alæ nasi et oris*, and the buceinator muscle. In such cases electro-muscular contractility is either normal or increased to both kinds of current. As this form of facial palsy has a central origin, faradisation is not to be recommended. The palsy frequently disappears spontaneously, but where it continues troublesome some months after the attack, galvanization may be resorted to, and generally proves successful. In tumors of the corpus striatum and crus cerebri, and in diseases of the pons, facial palsy appears combined with paralysis of the third and other cerebral nerves. In these cases the constant electric current cautiously applied, may relieve certain symptoms of the affection.

Facial palsy ensuing in the course of *locomotor ataxy* is generally an unfavorable sign, as it shows that the disease is gradually advancing towards the medulla oblongata, and the roots and nuclei of the cerebral nerves. The constant current may however, under the circumstances, be employed with a fair chance of temporary benefit.

Illustrative Cases.—Peripheral Paralysis.—As a boy 1 well remember riding to and from school a distance of several miles, during such a low degree of temperature as to produce peripheral paralysis of the face, which yielded to the usual treatment of friction and heat.

Central Paralysis.—No. 2. Is a case in my early practice of a man who suffered from a tumor of the base of the brain. When this gentleman elevated his face to shave, or for any other purpose, he immediately suffered facial paralysis; or, at this moment the light of the intellect would vanish from his face of expression. In case of his keeping his face upturned for any length of time he would suffer insensibility and fall to the floor.

Althaus mentions the case of a burrister, aged 35, having been exposed to a draught of cold air at a railway station, became affected with paralysis of the left portio dura. The physiognomical expression had entirely vanished from that side of the face. The patient was not able to laugh, frown, whistle, or shut his eye, which latter appeared staring and protruded. The angle of the mouth was depressed and drawn upon the opposite side; that of the sound side being higher and drawn towards the ear. The cheek was flabby and loose, and eating and drinking were troublesome. The patient was sent to Althaus by the late Dr. Todd, whom he had consulted six months after the commencement of the affection.

Faradi-muscular contractility was diminished. The paralysed muscles were individually faradised, with the effect that the patient regained his normal physiognomical expression after a fortnight's treatment.

65 North Charles Street. —*Medical Summary.*

